# Supplementary material for: Combined effect of probiotics and omega-3 fatty acids on blood and liver function biomarkers and gut microbiota diversity in male mice with high fat diet-induced obesity
Source: PLoS One. 2025 Sep 24;20(9):e0331981. doi: 10.1371/journal.pone.0331981 (PMC12459831; doi:10.1371/journal.pone.0331981)
Supplement: Attachment — Submitted filename: response to referees comments.docx [file pone.0331981.s002.docx]

# Response to Reviewers’ Comments

We sincerely thank the reviewers and the editor for their insightful and constructive comments. We have carefully revised the manuscript in accordance with their suggestions. Below, we provide a point-by-point response, indicating the changes made in the manuscript.

## Reviewer

**Comment 1 (Page 3):**

Comment: “Since omega- 3 cannot be synthesized in the body, absorption is only possible through food intake, so absorption rate is very important.” Suggest replacing with: 'can only be partly synthesized in the body, their availability…'

Response: Thank you for your suggestion. We have revised the sentence to reflect the fact that omega-3 fatty acids can be partly synthesized but are primarily obtained through diet.

Changes in the manuscript: “Since omega-3 fatty acids can only be partly synthesized in the body, their availability largely depends on dietary intake; therefore, the absorption rate is a critical factor in determining their physiological effectiveness.”

**Comment 2 (Page 4):**

Comment: The sentence regarding the study aim is unclear.

Response: We agree that the sentence could be improved for clarity and have revised it to clearly state the purpose of the study.

Changes in the manuscript: “This study aims to evaluate the synergistic effects of probiotics and omega-3 co-administration in a high-fat diet-induced obesity model by analyzing blood and liver function biomarkers, as well as to assess changes in gut microbiota diversity through microbiome analysis.”

**Comment 3 (Page 4):**

Comment: 300 mg/kg of omega-3 seems excessive; clarification of “60 kcal%” is also requested.

Response: We used the omega-3 dose based on precedent in previous studies, and no adverse effects were observed in our experimental animals. The “60% kcal” was an error and has been removed.

Changes in the manuscript: The incorrect “60% kcal” was deleted. Details of the diet composition were retained in the following sentence.

**Comment 4 (Page 5):**

Comment: Clarify frequency of food intake measurement.

Response: Both body weight and food intake were measured twice weekly. However, to avoid confusion, we removed the potentially misleading sentence.

Changes in the manuscript: The sentence “Food intake was calculated as the difference between the amount of food supplied the previous week and the remaining food.” has been deleted.

**Comment 5 (Page 5):**

Comment: Clarify whether animals were anesthetized or euthanized.

Response: Thank you. The correct term is “euthanized,” and we have updated the manuscript accordingly.

**Comment 6 (Page 5):**

Comment: Liver tissue samples were homogenized and processed, and optical density (OD) was measured at 570 nm using a microplate reader” difficult to understand.

Response: We have revised the sentence for clarity and to better describe the lipid quantification process.

Changes in the manuscript: “Liver tissues were first homogenized in an appropriate buffer to extract lipids. The homogenized samples were then subjected to the ELISA procedure, and the resulting reactions were quantified by measuring the optical density (OD) at 570 nm using a microplate reader.”

**Comment 7 (Page 6):**

Comment: Liver function improved only in TG, not all biomarkers.

Response: We agree. While statistical significance was found only in TG levels, the co-treatment group showed a trend toward improvement in total cholesterol. This has been appropriately discussed.

**Comment 8 (Page 7):**

Comment: The PT and aPTT of the HFD+DSF+omega3 group were 7.3±0.55 sec and 26.7±0.69 sec, respectively, which showed an improvement compared to the 7.0±0.29 sec and 25.1±1.80 sec of the HFD group,”; I have difficulty to understand because shorter time – quicker coagulation - mean a better situation (please to explain).

Response: Thank you for pointing this out. We clarified in the discussion that increased PT and aPTT times in the co-treatment group indicate inhibited thrombosis, suggesting a protective effect against coagulation-related risks.

**Comment 9 (Page 7):**

Comment: aPTT was longer in the co-treatment group, which could be interpreted as worse.

Response: As noted above, we interpret longer aPTT as indicating reduced coagulation activity, which may help prevent thrombosis. The discussion has been revised to reflect this interpretation.

**Comment 10 (Page 8):**

Comment: liver damage as they accumulate”; please to better specify what is accumulated.

Response: We revised the sentence to specifically refer to lipid accumulation as the cause of liver injury.

Changes in the manuscript: “In addition, elevated levels of cholesterol and triglycerides in the blood can lead to the accumulation of lipids within liver cells, which contributes to liver cell injury. This injury results in the release of liver enzymes such as AST and ALT into the bloodstream, thereby increasing their serum levels and serving as biomarkers of liver damage”

**Comment 11 (Page 8):**

Comment: Clarify how omega-3 absorption was evaluated.

Response: We did not directly measure absorption. The improved effects in the combination group suggest potential increased bioavailability. To avoid misunderstanding, we revised the text accordingly.

Changes in the manuscript: In addition, the aPTT, a blood coagulation test, showed a synergistic effect in the group receiving combined administration of omega-3 and probiotics, indicating improved blood coagulation time. While omega-3 is known to reduce lipid levels such as cholesterol in the blood and liver tissue, the enhanced efficacy observed in the combination group suggests that co-administration with probiotics may have improved omega-3 bioavailability, leading to greater physiological benefits.”

**Comment 12 (Page 8):**

Comment: Only ALT showed significant changes, not AST.

Response: Correct. We have clarified in the discussion that ALT showed a significant decrease in the co-treatment group, while AST showed only a downward trend.

**Comment 13 (Page 8):**

Comment: “In the triglycerides” – unclear expression.

Response: We have revised the sentence for clarity.

Changes in the manuscript: “Although a statistically significant synergistic effect of the combined administration was not confirmed for hepatic triglyceride levels, treatment with probiotics, omega-3, and their combination each demonstrated a trend toward improvement compared to the high-fat diet group”

**Comment 14 (Page 9):**

Comment: Insignificant AST and ALT results may not justify such strong conclusions.

Response: We appreciate this point. In the revised manuscript, we emphasized the significant ALT change and described the AST results as a trend.

**Comment 15 (Page 9):**

Comment: aPTT result seems to imply increased coagulation risk, not improvement.

Response: We clarified that prolonged aPTT in the co-treatment group indicates delayed coagulation, which can reduce the risk of thrombosis. This is now clearly explained in both the results and conclusion sections.
