# Supplementary material for: Combined effect of probiotics and omega-3 fatty acids on blood and liver function biomarkers and gut microbiota diversity in male mice with high fat diet-induced obesity
Source: PLoS One. 2025 Sep 24;20(9):e0331981. doi: 10.1371/journal.pone.0331981 (PMC12459831; doi:10.1371/journal.pone.0331981)
Supplement: Attachment — Submitted filename: Response to Reviewers.docx [file pone.0331981.s003.docx]

# Response to Reviewers

We sincerely thank the editor and the reviewers for their insightful and constructive comments. We have carefully revised the manuscript in accordance with their suggestions. Below, we provide a point-by-point response, indicating the changes made in the manuscript.

## Editorial Comments

**1. PLOS ONE style requirements and file naming**

We have revised the manuscript and submission files to comply with the PLOS ONE style requirements, including appropriate file naming.

**2. Clarification of euthanasia method**

We have revised the Methods section to clarify the process: mice were anesthetized using isoflurane, followed by blood collection and then euthanasia via cervical dislocation.

Change in the manuscript:

After 6 weeks of treatment, the mice were anesthetized with isoflurane (Hana Pharm, Co., Ltd., Seoul, Korea), and blood was collected. Thereafter, the mice were euthanized by cervical dislocation, and the liver was collected.

**3. Removal of funding information from manuscript body**

We have removed all funding-related statements from the manuscript body as instructed.

**4. Role of funders**

We have added the following statement to the cover letter as requested:

"The funders had no role in study design, data collection and analysis, decision to publish, or preparation of the manuscript."

**5. Data Availability**

We confirm that all minimal data required to replicate our findings are provided either within the manuscript or as Supporting Information files, including raw data for statistical analyses and figures.

**6. Reference list corrections**

We reviewed and corrected the entire reference list. All citations are now properly formatted, and no retracted articles are cited.

**7. Correction of sequence information error**

While reviewing the manuscript during revision, we identified an error in the description of a genetic sequence used in the 16S rRNA amplification protocol. Specifically, a portion of the primer sequence was incorrectly typed. We have corrected this sequence in the Methods section to ensure it accurately reflects the actual primers used in the experiment.

Change in the manuscript:

The corrected primer sequences for the V3–V4 region of the 16S rRNA gene are now accurately described as:

Forward primer:

5′- TCGTCGGCAGCGTAGGATGTGTATAAGAGACAGCCTACGGGNGCWGCAG

Reverse primer:

5′- GTCTCGTGGGCTCGGAGATGTGTATAAGAGACAGGACTACHVGGGTA-TCTAATCC

We apologize for this oversight and have ensured the information now matches the actual experimental procedures used.

## Reviewer #1

General Comment:

We thank Reviewer #1 for the positive assessment and thoughtful suggestions to improve clarity and accuracy.

**1. Taxonomic name formatting**

All taxonomic names have been revised to consistently use italic font and follow taxonomic conventions.

**2. Unsupported claims about DSF**

We have added appropriate references to support the claims regarding DSF’s effects on nutrient absorption, inflammation, and immune function (Line 72).

**3. Inconsistent referencing style**

All references and citation styles have been standardized throughout the manuscript. All cited works are now properly included in the reference list.

**4. Figure quality and missing axis labels**

We have added missing y-axis labels to Figure 5a and 5b:

- Figure 5a: “Relative Abundance (%)”

- Figure 5b: “Shannon Index”

We appreciate the reviewer’s suggestion regarding figure quality. In response, we have replaced the figures with higher-resolution versions to ensure improved clarity and readability in the revised manuscript.

**5. Misinterpretation of diversity indices**

To resolve the misunderstanding caused by the expression “richness,” the expression “richness” was deleted and unified as the alpha diversity index (Shanon index) throughout the manuscript.

**6. Multiple testing adjustment**

We confirm that statistical analyses were conducted using ANOVA or non-parametric tests, followed by post hoc analyses. Dunnett’s test was used for ANOVA, and Dunn’s test for non-parametric comparisons. These methods are described in the Statistical Analysis section.

We also performed FDR-adjusted p-value calculations (q-values) during differential abundance analysis, these were not included in the manuscript because most of the taxa of interest did not meet the adjusted significance threshold (FDR < 0.05). For instance, the adjusted p-values for the taxa shown in Figure 5a and 5b were 0.139 and 0.086, respectively. Given the exploratory nature of the study, we focused on unadjusted p-values to identify potentially meaningful taxa that may otherwise be overlooked under strict correction.

**7. Beta diversity analysis**

In response, we performed pairwise PERMANOVA tests (10,000 permutations) using Bray–Curtis dissimilarity to assess differences in microbial community composition. The analysis revealed significant differences between the HFD group and the other treatment groups: HFD vs. HFP: p = 0.013; HFD vs. HFO: p = 0.002; HFD vs. HFC: p = 0.001. These results further support the conclusion that the microbial communities in each treatment group are distinct from those in the HFD group. We have incorporated these pairwise comparisons into the Results section (Beta Diversity subsection) and noted this addition in the legend of Figure 5 in the revised manuscript.

## Reviewer #2

We sincerely thank Reviewer #2 for the encouraging and supportive comments. We are pleased to know that the study design, results, and interpretation were clear and well-aligned with the objectives.

## Reviewer #3

We appreciate Reviewer #3’s time and thoughtful feedback, even though the manuscript did not meet expectations. We will strive to improve the quality of future research and hope to share better findings in subsequent submissions.

## Reviewer #4

We thank Reviewer #4 for the positive evaluation and helpful comments to improve clarity and scientific rigor.

**1. Use of the term "synergistic"**

We agree with the suggestion. The term “synergistic” has been replaced with “combined” throughout the manuscript.

**2. ALT vs. AST clarity**

We clarified that only ALT showed a significant difference, while AST exhibited a non-significant downward trend.

Change in the manuscript:

Abstrat:

Over six weeks, the combined treatment group showed significant reductions in body weight gain, a significant improvement in ALT levels (a key liver function biomarker), and enhanced anticoagulation markers, such as prothrombin time and activated partial thromboplastin time, compared to the HFD group.

Results (Liver function section):

We also updated the Results section accordingly:

Regarding AST and ALT levels, the HFD+Co-treat group showed a significant decrease in ALT levels compared to the HFD group, while AST levels showed a non-significant decreasing trend.

**3. Use of only male mice**

We used only male mice to minimize biological variability caused by the estrous cycle in female mice, which can affect metabolic and microbiota parameters. This approach was chosen to ensure statistical power and consistency given the limited sample size.

**4. Caloric intake/energy expenditure differences**

As shown in Figure 1, food intake was higher in the omega-3 treated groups (HFD+Omega3 and HFD+Co-treat). However, food efficiency ratios (FER) were lower in these groups, suggesting more efficient metabolism or altered energy expenditure despite higher intake.

**5. Baseline microbiota profiles**

We did perform an analysis that included baseline samples; however, the results did not reveal any meaningful or interpretable patterns relevant to the subsequent changes observed after the interventions. Therefore, we did not include these data in the main manuscript. However, in response to the reviewer’s suggestion, we have added a figure showing baseline profiles for reference (Fig 1).

Fig 1. Principal coordinates analysis (PCoA) of gut microbiota based on Bray-Curtis dissimilarity. The left panel shows the overall microbial community structures of all groups before (Pre) and after (Post) treatment. The upper right panel displays Pre-treatment samples only, and lower right panel shows Post-treatment samples only. Groups include ND (normal diet), HF (high-fat diet), HFP (HF + probiotics), HFO (HF + omega-3), and HFC (HF + Probiotics + omega-3). Each dot represents an individual sample; ellipses indicate the 95% confidence interval for each group. Pairwise PERMANOVA p-values are summarized in the table.

**6. Omega-3 absorption and dose justification**

This study did not include direct measurement of plasma or tissue DHA/EPA concentrations. While this limits the ability to confirm bioavailability or absorption levels of omega-3 fatty acids, the study aimed primarily to evaluate physiological outcomes such as metabolic biomarkers and gut microbiota diversity. Future investigations should incorporate quantitative analysis of DHA/EPA levels to clarify their correlation with observed physiological effects.

Regarding dose: The 300 mg/kg dose of omega-3 fatty acids used in this study corresponds to an approximate human equivalent dose (HED) of 1.45 g/day, which falls within the range of doses commonly employed in clinical studies for hyperlipidemia or cardiovascular support. This dose was therefore considered physiologically relevant for evaluating potential therapeutic effects in a high-fat diet-induced obesity model.

**7. Statistical assumptions**

We confirm that normality and log-normality tests were performed prior to conducting ANOVA or non-parametric tests.

**8. Figure legends and error bars**

All figures now use standard deviation (SD) for error bars. We have removed mentions of SEM in the manuscript for consistency.

**9. Raw 16S data availability**

The Desimone formulation used in this study is a proprietary probiotic blend composed of eight different strains. There is a possibility that the 16S rRNA sequencing results may contain partial or full sequences of these strains. In accordance with our internal policy and intellectual property protection regulations, we are unable to publicly release raw sequencing data that may disclose strain-specific genomic information. We respectfully ask for your understanding regarding the exclusion of raw 16S rRNA data from public repositories under these circumstances.

**10. Grammar corrections**

We have revised the manuscript to improve grammar and phrasing for better clarity and readability.
