# Supplementary material for: Combined effect of probiotics and omega-3 fatty acids on blood and liver function biomarkers and gut microbiota diversity in male mice with high fat diet-induced obesity
Source: PLoS One. 2025 Sep 24;20(9):e0331981. doi: 10.1371/journal.pone.0331981 (PMC12459831; doi:10.1371/journal.pone.0331981)
Supplement: Attachment — Submitted filename: Response_to_Reviewers_2nd revised.docx [file pone.0331981.s004.docx]

# Response to Reviewers (2nd Revision)

We sincerely thank the editor and reviewers for their thoughtful and constructive comments. We have carefully revised the manuscript based on the remaining concerns. Below, we provide our detailed responses to each point, along with the changes made in the manuscript.

## Reviewer #1

**1. Taxonomic naming**

We appreciate the reviewer for pointing this out. Although taxonomic names were italicized during the previous revision, it appears that the formatting was not preserved during the PDF conversion process. We have now double-checked and ensured that all taxonomic names are correctly italicized in the final version.

Additionally, the term “thermopiles” was indeed a typographical error and has been corrected to Streptococcus thermophilus.

**2. References supporting DSF claims**

Because it was difficult to find supporting references beyond Reference 16 (related to probiotics and autism), we removed the autism-related content from the main text. Reference 17 has also been replaced with a more appropriate source.

**3. Referencing accuracy and formatting**

We have reviewed and corrected the mentioned references. The DOI in Reference 19 has been updated to link to the correct article, and Reference 20 has been corrected to Amir et al., as appropriate.

## Reviewer #4

**1. Data availability and reproducibility**

In accordance with the reviewer’s recommendation, we have deposited the 16S rRNA sequencing data in the NCBI database.

Submission ID: SUB15518308

BioProject ID: PRJNA1302007

**2. Use of male-only mice and overgeneralization to humans**

We appreciate this important observation. As recommended:

- The manuscript title has been revised to indicate that the study was conducted in male mice only.

Revised Title:

Combined effect of probiotics and omega-3 fatty acids on blood and liver function biomarkers and gut microbiota diversity in male mice with high fat diet-induced obesity

- The language throughout the manuscript has been revised to avoid generalizations to all humans. Terms such as “human” were replaced with more precise phrases like “male” or “male mice” where appropriate.

- We have also added a statement to the Discussion section acknowledging the limitation of using male mice exclusively and highlighting the need for sex-inclusive research in future studies.

Change in the manuscript:

This study was conducted exclusively in male mice, which limits the generalizability of the findings to both sexes. As metabolic responses can differ significantly between sexes due to hormonal and physiological factors, future research including both male and female animals is necessary to validate and extend these findings. Future studies could further investigate the mechanistic pathways involved and explore the long-term clinical applications of these findings, with careful consideration of sex differences in human populations.
